# A Case of Prolonged Delayed Postdural Puncture Headache in a Patient with Multiple Sclerosis Exacerbated by Air Travel

**DOI:** 10.1155/2013/253218

**Published:** 2013-01-22

**Authors:** Jahan Porhomayon, Gino Zadeii, Alireza Yarahamadi, Nader D. Nader

**Affiliations:** ^1^VA Western New York Healthcare System, Division of Critical Care and Pain Medicine, Department of Anesthesiology, School of Medicine and Biomedical Sciences, State University of New York at Buffalo, Buffalo, NY, USA; ^2^VA Medical Center, Rm 203C, 3495 Bailey Ave, Buffalo, NY 14215, USA; ^3^Mason City Cardiology Clinic, University of Iowa, Mason City, IA 50401, USA; ^4^Mason City Neurology Clinic, University of Iowa, Mason City, IA 50401, USA; ^5^VA Western New York Healthcare System, Division of Cardiothoracic Anesthesia and Pain Medicine, Department of Anesthesiology, School of Medicine and Biomedical Sciences, State University of New York at Buffalo, Buffalo, NY 14215, USA

## Abstract

The developments of new spinal needles and needle tip designs have reduced the incidence of postdural puncture headache (PDPH). Although it is clear that reducing the loss of CSF leak from dural puncture reduces the headache, there are areas regarding the pathogenesis, treatment, and prevention of PDPH that remain controversial. Air travel by itself may impose physiological alteration in central nervous system that may be detrimental to patients with PDPH. This case report highlights a case of a young female patient who suffered from a severe incapacitating PDPH headache during high-altitude flight with a commercial jet.

## 1. Introduction

The first case report of postdural puncture headache (PDPH) was described in about 100 years ago by Bier and his assistant [1]. It was later postulated that PDPH is triggered by leakage of cerebrospinal fluid through the dural rent, but the cause of the pain is probably due to intracranial arterial and venous dilatation [2]. PDPH remains one of the major complications of spinal tap performed for diagnostic purposes. Other adverse events after lumbar puncture include dysesthesia, backache, nerve palsies, infectious processes, and bleeding disorders [3]. The patterns of development of PDPH depend on a number of procedure and nonprocedure-related risk factors. Knowledge of procedure-related factors supports interventions designed to reduce the incidence of PDPH. Despite the best preventive efforts, PDPH may still occur and be associated with significant morbidity [4, 5]. The potential risks for developing PDPH include female gender [6], young adults, repeated attempt with multiple dural punctures, and the size/type and orientation of the needle [7]. Gender is believed to be an independent risk factor for the development of PDPH as demonstrated by the recent meta-analysis by Wu et al. [6]. Clinical presentation of the PDPH or “spinal headache” is usually described as a severe, dull pain, usually frontal occipital, which is irritated in the upright position and decreased in the supine position. It may or may not be accompanied by nausea, vomiting, and visual/auditory disturbances. The onset of PDPH is between 2 to 72 hours, and latency period of up to 15 days has generally been described in the literature [8, 9]. 

## 2. Case Report

**Figure 1:**
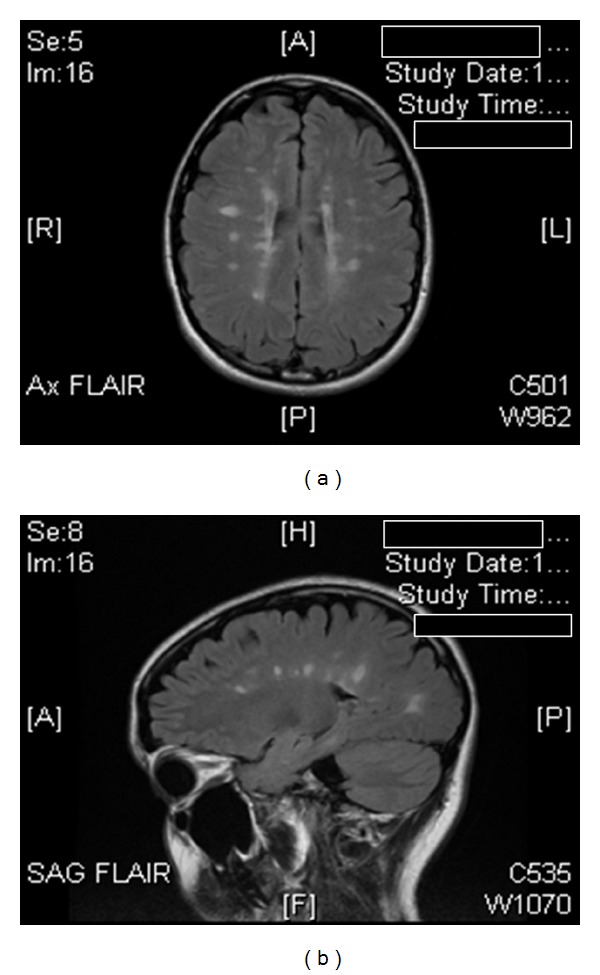
R: imaging demonstrates periventricular high signal intensity lesions, which exhibit a typical distribution for multiple sclerosis (Dawson's fingers). A: it shows corpus callosal hyperintensities suggestive of a demyelinating process. Patient does not have evidence of brain stem sagging, dilatation of vein or dural sinuses, and effacement of hemispheric cortical sulci. Absence of any of these signs does not preclude the diagnosis of postdural puncture headache.

This is unique case of a young 23 years old middle Eastern female who developed an acute unilateral eye pain and generalized headache with visual disturbances associated with fatigue and weakness in lower extremities for two days. She presented to a local community hospital and was examined by a neurologist. Physical examination revealed an exaggerated deep tendon reflexes and sustained clonus of extensor plantar responses. Ophthalmologic examination was normal. A spinal tap was performed in the neurologist office with a 22 gauge Quincke spinal needle between the third and fourth lumbar spaces after the first attempt with free flow of clear cerebral spinal fluid (CSF). This fluid was analyzed for IgG, albumin, and oligoclonal banding to confirm the diagnosis of multiple sclerosis (MS), and a magnetic resonance imaging (MRI) of the brain was also obtained. All laboratory tests confirmed the diagnosis of MS. Patient returned home with follow-up appointment in 2 days. She later developed severe headache 10 hours after returning home. Headache was more localized to the back of the head and worsened with ambulation. Patient returned to the neurologist office the next day and was prescribed acetaminophen with bed rest. Nevertheless, she continued with severe headache on ambulation. In addition, she experienced dizziness and neck stiffness for the next 7 days. Follow-up evolutions and repeat physical examination revealed similar findings. Patient family remained concerned and decided to travel to the USA for additional treatment and consultation. She completed an 18 hours flight from her home town to the USA in a sitting position. At the completion of her journey, she experienced severe occipital and frontal headache associated with nausea/vomiting with neck stiffness and back pain. The pain intensity increased particularly during aircraft takeoff. Upon landing at airport, she had to be transported by a wheel chair to her car. She was seen the next day by another neurologist in the USA and after a complete examination was diagnosed with PDPH and repeated MRI of brain was completed at the same day (Figure 1). She was prescribed complete bed rest, oral analgesics, caffeine 300 mg orally. She was seen again in 72 hours with similar complain of occipital headache worse on ambulation and was referred to a pain clinic for epidural blood patch. An appointment was scheduled for her with an anesthesiologist 4 weeks later after obtaining insurance coverage. An epidural blood patch was performed by withdrawing 20 mL of blood from the right antecubital vein under aseptic condition. At the same, the epidural space was identified between the third and fourth lumbar vertebrate space using the loss of resistance technique. Subsequently, 20 mL of blood was injected into the epidural space. She had an immediate relief of her headache and was able to ambulate to her car without difficulty.

## 3. Discussion

Here we report a case of PDPH from spinal tab for the evaluation of MS that lasted up to 6 weeks after procedure and was exacerbated by air travel and prolong sitting position. There is scarce data on prolonged case of PDPH and the physiologic impact of air travel on regional spinal and intracranial pressures following dural puncture. 

Despite the current recommendations for the use of atraumatic and smaller size spinal catheters for diagnostic spinal puncture [10], we continue to see reported cases of PDPH in the literatures worldwide [11]. Pedersen reported 42% incidence of PDPH with a 22 Quincke needle in patient undergoing diagnostic lumbar punctures [12]. The study by Hammond et al. reported the incidence of PDPH at 30 percents with the use of traumatic 20 and 22 gauges Quincke spinal needles [7]. Geurts et al. reported an incidence of 25% PDPH in a prospective study of 80 patients less than 40 years of age, given spinal anesthesia through a 0.52 mm (25 gauge) needle [13]. The incidence of PDPH after lumbar puncture can be reduced from 36% to 0–9% with the use of an atraumatic needle size 24 gauge (G)/0.56 mm rather than a traumatic needle size 22 G/0.7 mm [11]. As a result, selection of a spinal needle played a major role for developing PDPH. Additionally, air travel probably contributed to exacerbation of her symptoms. There is only one previously published case report of prolonged PDPH after air travel [14]. Vacanti reported a case of an 18-year-old soldier who developed PDPH after a knee surgery. He had increased headache with air travel 7 days after operation. The headache intensified particularly during takeoff and subsided several days later [15]. Mulroy reported an unintentional dural puncture postepidural anesthesia that was treated with epidural blood patch twice. His patient developed PDPH after air travel that lasted up to six weeks and relieved with epidural blood patch [14]. Likewise, Panadero et al. reported a case of a healthy 42-year-old adult male who underwent arthroscopy with spinal anesthesia and was completely asymptomatic. Thirty-six hours after procedure and 10 minutes after aircraft took off, he complained of severe headache and nausea. Symptoms subsided after landing and resolved few days later [16]. It was postulated that rapid decompression during aircraft taking off may have altered the relationship of epidural and dural pressures and resulted in increased CSF leak during landing or taking off. Additionally, autoregulatory changes of CSF pressure at high altitudes may have been impaired [17]. In 1962 Safar and Tenicela also reported increased incidence of PDPH at higher altitudes when anesthesiologist performed spinal anesthesia [18]. Data available from Singh et al. looking invasively at the lumbar CSF pressures in 34 Indian soldiers transported rapidly via helicopter from sea level to altitude of 5867 meters recorded elevated pressures of 6–20 cm H_2_O compared to baseline suggesting impaired CSF pressure regulation with rapid ascend [19]. Other investigators have reported rise in intracranial pressure secondary to hypoxia rise in intracranial pressure secondary to hypoxia [20, 21]. Therefore, in our patient a combination of factors may have contributed to severe PDPH. Not only size and type of needle played an important role in developing PDPH but also air travel, high altitude, hypoxia, changes in CSF and intracranial pressure, and prolonged sitting position may have all contributed to worsening of her symptoms.

Our current knowledge regarding air travel and physiologic changes related to spinal and epidural pressures is very limited. Moreover, autoregulatory mechanisms for maintaining CSF pressures are disturbed during high-altitude flights. Further studies are needed to provide the clinicians with recommendations and management of PDPH on long-range intercontinental flights after dural puncture or central neuraxial anesthesia.
